# 1,2-*O*-Iso­propyl­idene-β-d-*lyxo*-furan­ose

**DOI:** 10.1107/S2414314620016302

**Published:** 2020-12-22

**Authors:** Bogdan Doboszewski, Alexander Y. Nazarenko

**Affiliations:** a Departamento de Química, Universidade Federal Rural de Pernambuco, 52171-900 Recife, PE, Brazil; b Chemistry Department, State University of New York, College at Buffalo, 1300 Elmwood Ave, Buffalo, NY 14222-1095, USA; University of Zürich, Switzerland

**Keywords:** crystal structure, carbohydrate, furan­ose

## Abstract

In the title compound C_8_H_14_O_5_, the pento­furan­ose five-membered ring has a twisted conformation on two carbon atoms while the five-membered ring of the iso­propyl­idene group has an envelope conformation on an oxygen atom. Hy­droxy groups are involved an infinite network of O—H⋯O hydrogen bonds that leads to the formation of a layer parallel to the (001) plane. Only weak C—H⋯O contacts exist between neighboring layers.

## Abstract 2

In the title compound, the pento­furan­ose ring has a twisted conformation while the other five-membered ring has an envelope conformation; the two hy­droxy groups C are involved in an infinite network of O—H⋯ O bonds, forming a layer parallel to the (001) plane.

## Graphical abstract

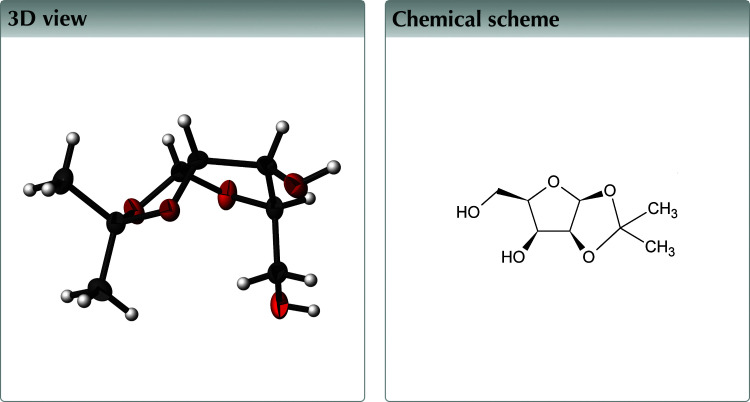

## Structure description

The title compound, C_8_H_14_O_5_, (**1**) together with its enanti­omeric *L* form, are relatively rare derivatives and a limited volume of information is available for either of them. Our inter­est in **1** stems from the possibility of conducting de­oxy­genation at its C3 position to obtain 3-de­oxy-1,2-*O*-iso­propyl­idene-β-d-*threo-*pento­furan­ose as a chiral synthon for further synthetic work (Soares *et al.*, 2013 ▸). Compound **1** was obtained from the known 1,2-*O*-iso­propyl­idene-5-*O*-*t-*butyl­diphenyl­silyl-β-d-*arabino*-furan­ose **2** (Dahlman *et al.*, 1986 ▸) *via* oxidation at the C3 position followed by reduction of the inter­mediate ulose. The reduction proceeded with a total stereoselection from the more accessible *Re* (α) side to furnish 1,2-*O*-iso­propyl­idene-5-*O*–*t-*butyl­diphenyl­silyl-β-d-*lyxo*-furan­ose, whose desilylation gave the target **1**. It should be pointed out that under these iso­propyl­idenation conditions, d-lyxose furnished only its α,β-2,3-*O*-iso­propyl­idene­furan­ose (Barbat *et al.*, 1991 ▸). Compound **1** was previously obtained starting from d-glucose *via* d-gulose (Kuzuhara *et al.*, 1971 ▸). The scarcity of any experimental data on **1** prompted us to examine its structure.

**Figure 1:**
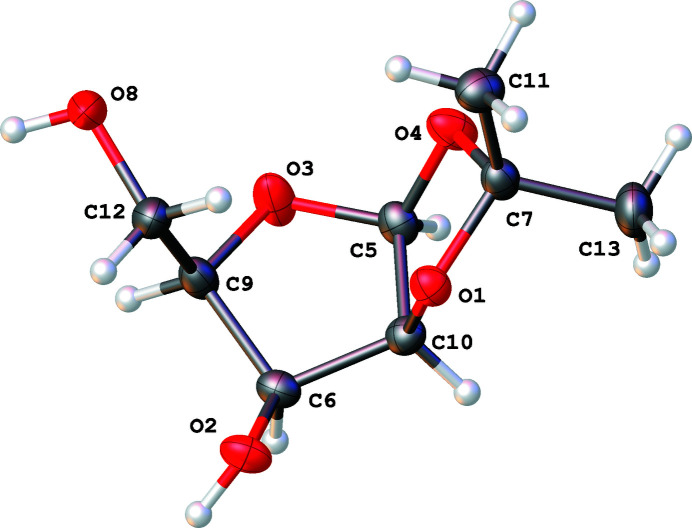
The title compound with the atom-numbering scheme and 50% probability displacement ellipsoids

In the title compound (Fig. 1 ▸), the pento­furan­ose five-membered ring has twisted conformation on atoms C6 and C9 [*Q* = 0.3175 (12) Å, φ = 117.6 (2)°]. The five-membered ring of the iso­propyl­idene group has an envelope conformation on atom O1 [Q(2) = 0.3192 (11) Å, φ = 187.1 (2)°]. Puckering parameters (Cremer & Pople, 1975 ▸) were calculated using *PLATON* (Spek, 2020 ▸). We have observed the same conformation of the iso­propyl­edene fragment in other carbohydrates (Doboszewski & Naza­renko, 2003 ▸; Doboszewski *et al.*, 2010 ▸).

**Figure 2:**
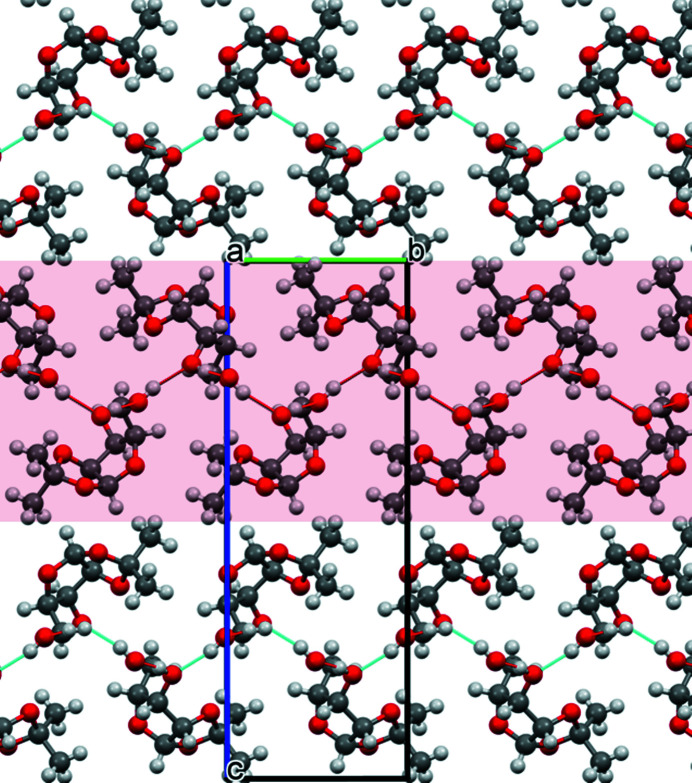
Packing diagram of the title compound; view along [100] vector. Highlighted are the layers of mol­ecules connected *via* O—H⋯O hydrogen bonds.

**Figure 3:**
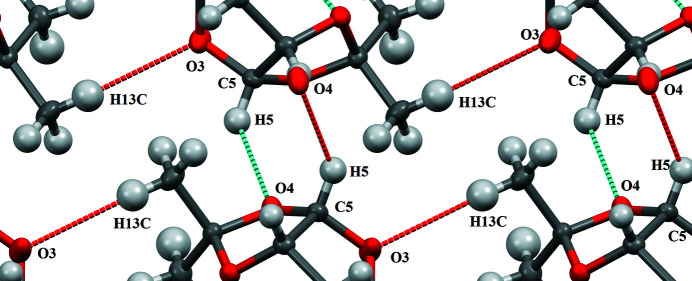
Detail view of the inter­molecular C—H⋯O inter­actions.

In the crystal, the two hy­droxy groups form an infinite network of O—H⋯O hydrogen bonds that leads to the formation of a layer parallel to the (001) plane (Table 1 ▸, Fig. 2 ▸). Only weak C—H⋯O (Fig. 3 ▸) contacts exist between neighboring layers; the C5⋯O4(− 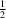 + *x*, 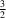 − *y*, −*z*) distance is 3.389 (2) Å. Similar hydrogen bonds have been observed in various carbohydrates (Desiraju & Steiner, 1999 ▸). A short intra­molecular contact between oxygen O1 and the H12*B* atom of a neighboring methyl­ene group (Table 1 ▸) may additionally stabilize the conformation of the mol­ecule. Therefore, all oxygen atoms of the title mol­ecule participate in O—H⋯O or C—H⋯O inter­actions.

## Synthesis and crystallization

The synthesis of the title compound is described in Kuzuhara *et al.* (1971 ▸) and Soares *et al.* (2013 ▸).

## Refinement

Crystal data, data collection and structure refinement details are summarized in Table 2 ▸. An additional dataset was collected using Cu *K*α radiation, resulting in a Flack parameter of 0.09 (13) and a probability of the absolute configuration being correct of 1.000.

## Supplementary Material

Crystal structure: contains datablock(s) I. DOI: 10.1107/S2414314620016302/zq4044sup1.cif

Structure factors: contains datablock(s) I. DOI: 10.1107/S2414314620016302/zq4044Isup2.hkl

Supporting information file. DOI: 10.1107/S2414314620016302/zq4044Isup3.cdx

CuK(alpha) CIF file for chirality confirmation. DOI: 10.1107/S2414314620016302/zq4044sup4.txt

CCDC reference: 2050681

Additional supporting information:  crystallographic information; 3D view; checkCIF report

## Figures and Tables

**Table 1:** Hydrogen-bond geometry (Å, °)

*D*—H⋯*A*	*D*—H	H⋯*A*	*D*⋯*A*	*D*—H⋯*A*
O2—H2⋯O8^i^	0.88 (2)	1.72 (2)	2.5946 (15)	169 (2)
O8—H8⋯O2^ii^	0.84 (2)	1.86 (2)	2.6567 (16)	158 (2)
C12—H12*B*⋯O1	0.95 (2)	2.54 (2)	3.1908 (15)	126.1 (14)

Symmetry codes: (i) 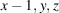 ; (ii) 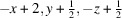 .

**Table 2:** Experimental details

Crystal data	
Chemical formula	C_8_H_14_O_5_
*M*_r_	190.19
Crystal system, space group	Orthorhombic, *P*2_1_2_1_2_1_
Temperature (K)	173
*a*, *b*, *c* (Å)	5.9196 (3), 7.3562 (4), 21.1126 (12)
*V* (Å^3^)	919.36 (9)
*Z*	4
Radiation type	Mo *K*α
μ (mm^−1^)	0.12
Crystal size (mm)	0.45 × 0.43 × 0.37

Data collection	
Diffractometer	Bruker PHOTON-100 CMOS
Absorption correction	Multi-scan (*SADABS*; Krause *et al.*, 2015 ▸)
*T*_min_, *T*_max_	0.944, 1.000
No. of measured, independent and observed [*I* > 2σ(*I*)] reflections	20693, 4938, 4094
*R*_int_	0.029
(sin θ/λ)_max_ (Å^−1^)	0.862

Refinement	
*R*[*F* ^2^ > 2σ(*F* ^2^)], *wR*(*F* ^2^), *S*	0.043, 0.109, 1.03
No. of reflections	4938
No. of parameters	174
H-atom treatment	All H-atom parameters refined
Δρ_max_, Δρ_min_ (e Å^−3^)	0.41, −0.30
Absolute structure	Flack *x* determined using 1496 quotients [(*I* ^+^)−(*I* ^−^)]/[(*I* ^+^)+(*I* ^−^)] (Parsons *et al.*, 2013 ▸)
Absolute structure parameter	0.3 (2) su large for Mo Kα?

Computer programs: *APEX2* and *SAINT* (Bruker, 2013 ▸), *SHELXT* (Sheldrick, 2015*a* ▸), *SHELXL* (Sheldrick, 2015*b* ▸), *OLEX2* (Dolomanov *et al.*, 2009 ▸) and *Mercury* (Macrae *et al.*, 2020 ▸).

## full crystallographic data

### Crystal data

**Table:**

C_8_H_14_O_5_	*D*_x_ = 1.374 Mg m^−^^3^
*M**_r_* = 190.19	Mo *K*α radiation, λ = 0.71073 Å
Orthorhombic, *P*2_1_2_1_2_1_	Cell parameters from 9980 reflections
*a* = 5.9196 (3) Å	θ = 2.9–37.3°
*b* = 7.3562 (4) Å	µ = 0.12 mm^−^^1^
*c* = 21.1126 (12) Å	*T* = 173 K
*V* = 919.36 (9) Å^3^	Block, colourless
*Z* = 4	0.45 × 0.43 × 0.37 mm
*F*(000) = 408	

### Data collection

**Table:**

Bruker PHOTON-100 CMOS diffractometer	4938 independent reflections
Radiation source: sealed tube	4094 reflections with *I* > 2σ(*I*)
Graphite monochromator	*R*_int_ = 0.029
Detector resolution: 10.4 pixels mm^-1^	θ_max_ = 37.8°, θ_min_ = 2.9°
φ and ω scans	*h* = −9→10
Absorption correction: multi-scan (SADABS; Krause *et al.*, 2015)	*k* = −12→12
*T*_min_ = 0.944, *T*_max_ = 1.000	*l* = −27→36
20693 measured reflections	

### Refinement

**Table:**

Refinement on *F*^2^	Secondary atom site location: difference Fourier map
Least-squares matrix: full	Hydrogen site location: difference Fourier map
*R*[*F*^2^ > 2σ(*F*^2^)] = 0.043	All H-atom parameters refined
*wR*(*F*^2^) = 0.109	*w* = 1/[σ^2^(*F*_o_^2^) + (0.0654*P*)^2^ + 0.0319*P*] where *P* = (*F*_o_^2^ + 2*F*_c_^2^)/3
*S* = 1.03	(Δ/σ)_max_ < 0.001
4938 reflections	Δρ_max_ = 0.41 e Å^−^^3^
174 parameters	Δρ_min_ = −0.30 e Å^−^^3^
0 restraints	Absolute structure: Flack *x* determined using 1496 quotients [(*I*^+^)-(*I*^-^)]/[(*I*^+^)+(*I*^-^)] (Parsons *et al.*, 2013)
Primary atom site location: dual	Absolute structure parameter: 0.3 (2)

### Special details

**Table:**

Geometry. All esds (except the esd in the dihedral angle between two l.s. planes) are estimated using the full covariance matrix. The cell esds are taken into account individually in the estimation of esds in distances, angles and torsion angles; correlations between esds in cell parameters are only used when they are defined by crystal symmetry. An approximate (isotropic) treatment of cell esds is used for estimating esds involving l.s. planes.
Refinement. All hydrogen atoms are refined in isotropic approximation.

### Fractional atomic coordinates and isotropic or equivalent isotropic displacement parameters (Å^2^)

**Table:**

	*x*	*y*	*z*	*U*_iso_*/*U*_eq_	
O1	0.94830 (15)	0.59218 (11)	0.12758 (4)	0.02153 (15)	
O2	0.66312 (15)	0.80371 (13)	0.20056 (4)	0.02597 (18)	
H2	0.556 (4)	0.878 (3)	0.2138 (10)	0.044 (6)*	
O3	1.1047 (2)	0.99300 (12)	0.10435 (5)	0.0312 (2)	
O4	1.21881 (17)	0.71302 (12)	0.06429 (5)	0.02782 (19)	
O8	1.31943 (18)	1.00933 (18)	0.22671 (6)	0.0398 (3)	
H8	1.294 (4)	1.090 (3)	0.2540 (11)	0.044 (6)*	
C5	1.0438 (2)	0.84268 (15)	0.06643 (5)	0.02228 (19)	
H5	1.021 (4)	0.887 (3)	0.0247 (9)	0.034 (5)*	
C6	0.7654 (2)	0.88480 (15)	0.14745 (5)	0.02169 (19)	
H6	0.661 (3)	0.966 (3)	0.1270 (9)	0.029 (5)*	
C7	1.1331 (2)	0.54276 (15)	0.08756 (5)	0.02213 (19)	
C9	0.9802 (2)	0.99245 (15)	0.16300 (5)	0.0234 (2)	
H9	0.946 (3)	1.122 (2)	0.1724 (8)	0.021 (4)*	
C10	0.8456 (2)	0.74699 (15)	0.09853 (5)	0.02134 (18)	
H10	0.728 (4)	0.710 (3)	0.0702 (8)	0.029 (5)*	
C11	1.3131 (2)	0.45089 (18)	0.12639 (7)	0.0291 (2)	
H11A	1.446 (5)	0.431 (4)	0.0981 (11)	0.057 (7)*	
H11B	1.367 (4)	0.524 (3)	0.1593 (11)	0.039 (5)*	
H11C	1.264 (4)	0.337 (3)	0.1401 (9)	0.040 (5)*	
C12	1.1131 (2)	0.91394 (18)	0.21779 (6)	0.0272 (2)	
H12A	1.027 (4)	0.919 (3)	0.2536 (9)	0.027 (4)*	
H12B	1.143 (3)	0.789 (3)	0.2097 (9)	0.030 (5)*	
C13	1.0527 (3)	0.4265 (2)	0.03239 (7)	0.0335 (3)	
H13A	0.942 (4)	0.487 (3)	0.0070 (10)	0.038 (5)*	
H13B	1.178 (4)	0.399 (3)	0.0052 (11)	0.039 (5)*	
H13C	0.997 (4)	0.314 (3)	0.0495 (11)	0.048 (6)*	

### Atomic displacement parameters (Å^2^)

**Table:**

	*U*^11^	*U*^22^	*U*^33^	*U*^12^	*U*^13^	*U*^23^
O1	0.0226 (4)	0.0202 (3)	0.0218 (3)	−0.0006 (3)	0.0036 (3)	0.0012 (3)
O2	0.0209 (4)	0.0287 (4)	0.0283 (4)	0.0024 (3)	0.0084 (3)	0.0056 (3)
O3	0.0406 (5)	0.0230 (4)	0.0302 (4)	−0.0094 (4)	0.0161 (4)	−0.0036 (3)
O4	0.0254 (4)	0.0216 (3)	0.0364 (4)	0.0009 (3)	0.0104 (3)	0.0034 (3)
O8	0.0203 (4)	0.0466 (6)	0.0527 (6)	0.0001 (4)	0.0012 (4)	−0.0295 (5)
C5	0.0271 (5)	0.0219 (4)	0.0178 (4)	0.0010 (4)	0.0032 (4)	0.0018 (3)
C6	0.0197 (4)	0.0237 (4)	0.0216 (4)	0.0030 (4)	0.0022 (3)	0.0031 (4)
C7	0.0245 (5)	0.0190 (4)	0.0229 (4)	−0.0015 (3)	0.0036 (4)	−0.0009 (3)
C9	0.0254 (5)	0.0200 (4)	0.0248 (4)	−0.0010 (4)	0.0074 (4)	−0.0021 (4)
C10	0.0204 (4)	0.0242 (4)	0.0194 (4)	−0.0005 (3)	−0.0017 (3)	0.0006 (3)
C11	0.0261 (5)	0.0259 (5)	0.0351 (6)	0.0023 (4)	−0.0018 (5)	0.0018 (5)
C12	0.0228 (5)	0.0307 (5)	0.0280 (5)	−0.0011 (4)	−0.0006 (4)	−0.0080 (4)
C13	0.0401 (7)	0.0305 (6)	0.0298 (5)	0.0008 (5)	−0.0020 (5)	−0.0109 (5)

### Geometric parameters (Å, º)

**Table:**

O1—C7	1.4291 (14)	C6—C10	1.5230 (16)
O1—C10	1.4292 (14)	C7—C11	1.5049 (18)
O2—H2	0.88 (3)	C7—C13	1.5214 (17)
O2—C6	1.4071 (14)	C9—H9	0.993 (17)
O3—C5	1.4120 (14)	C9—C12	1.5135 (18)
O3—C9	1.4409 (14)	C10—H10	0.95 (2)
O4—C5	1.4090 (15)	C11—H11A	1.00 (3)
O4—C7	1.4380 (14)	C11—H11B	0.94 (2)
O8—H8	0.84 (3)	C11—H11C	0.93 (3)
O8—C12	1.4210 (17)	C12—H12A	0.91 (2)
C5—H5	0.947 (19)	C12—H12B	0.95 (2)
C5—C10	1.5267 (16)	C13—H13A	0.96 (2)
C6—H6	0.96 (2)	C13—H13B	0.96 (2)
C6—C9	1.5334 (17)	C13—H13C	0.96 (3)
			
C10—O1—C7	105.93 (8)	C12—C9—C6	113.43 (10)
C6—O2—H2	107.3 (14)	C12—C9—H9	108.6 (10)
C5—O3—C9	110.76 (9)	O1—C10—C5	103.36 (9)
C5—O4—C7	108.61 (9)	O1—C10—C6	111.84 (8)
C12—O8—H8	106.6 (17)	O1—C10—H10	110.7 (12)
O3—C5—H5	107.2 (12)	C5—C10—H10	114.3 (11)
O3—C5—C10	107.79 (9)	C6—C10—C5	103.51 (9)
O4—C5—O3	111.14 (10)	C6—C10—H10	112.6 (12)
O4—C5—H5	107.7 (13)	C7—C11—H11A	107.4 (15)
O4—C5—C10	105.50 (9)	C7—C11—H11B	112.8 (14)
C10—C5—H5	117.5 (14)	C7—C11—H11C	110.7 (13)
O2—C6—H6	110.1 (12)	H11A—C11—H11B	105 (2)
O2—C6—C9	113.91 (9)	H11A—C11—H11C	107 (2)
O2—C6—C10	113.10 (9)	H11B—C11—H11C	113.2 (18)
C9—C6—H6	107.9 (12)	O8—C12—C9	111.08 (11)
C10—C6—H6	108.1 (12)	O8—C12—H12A	110.5 (13)
C10—C6—C9	103.33 (9)	O8—C12—H12B	110.0 (12)
O1—C7—O4	104.51 (8)	C9—C12—H12A	109.2 (12)
O1—C7—C11	109.53 (10)	C9—C12—H12B	109.1 (12)
O1—C7—C13	110.88 (10)	H12A—C12—H12B	106.9 (16)
O4—C7—C11	109.11 (10)	C7—C13—H13A	112.5 (13)
O4—C7—C13	109.77 (10)	C7—C13—H13B	109.5 (14)
C11—C7—C13	112.71 (11)	C7—C13—H13C	107.8 (13)
O3—C9—C6	103.98 (9)	H13A—C13—H13B	107.1 (18)
O3—C9—H9	105.9 (10)	H13A—C13—H13C	112 (2)
O3—C9—C12	113.08 (11)	H13B—C13—H13C	108 (2)
C6—C9—H9	111.5 (11)		
			
O2—C6—C9—O3	155.65 (9)	C6—C9—C12—O8	175.05 (9)
O2—C6—C9—C12	32.41 (13)	C7—O1—C10—C5	−31.62 (10)
O2—C6—C10—O1	−41.18 (13)	C7—O1—C10—C6	−142.34 (9)
O2—C6—C10—C5	−151.81 (9)	C7—O4—C5—O3	121.62 (10)
O3—C5—C10—O1	−102.57 (10)	C7—O4—C5—C10	5.06 (12)
O3—C5—C10—C6	14.19 (12)	C9—O3—C5—O4	−108.31 (11)
O3—C9—C12—O8	56.97 (13)	C9—O3—C5—C10	6.84 (13)
O4—C5—C10—O1	16.25 (11)	C9—C6—C10—O1	82.45 (11)
O4—C5—C10—C6	133.01 (9)	C9—C6—C10—C5	−28.18 (11)
C5—O3—C9—C6	−24.89 (13)	C10—O1—C7—O4	35.34 (11)
C5—O3—C9—C12	98.58 (12)	C10—O1—C7—C11	152.12 (9)
C5—O4—C7—O1	−24.63 (12)	C10—O1—C7—C13	−82.87 (11)
C5—O4—C7—C11	−141.71 (10)	C10—C6—C9—O3	32.55 (11)
C5—O4—C7—C13	94.33 (12)	C10—C6—C9—C12	−90.68 (11)

### Hydrogen-bond geometry (Å, º)

**Table:**

*D*—H···*A*	*D*—H	H···*A*	*D*···*A*	*D*—H···*A*
O2—H2···O8^i^	0.88 (2)	1.72 (2)	2.5946 (15)	169 (2)
O8—H8···O2^ii^	0.84 (2)	1.86 (2)	2.6567 (16)	158 (2)
C12—H12*B*···O1	0.95 (2)	2.54 (2)	3.1908 (15)	126.1 (14)

Symmetry codes: (i) *x*−1, *y*, *z*; (ii) −*x*+2, *y*+1/2, −*z*+1/2.
